# Effects of *Diaphorina citri* Population Density on Daily Timing of Vibrational Communication Calls: Potential Benefits in Finding Forage

**DOI:** 10.3390/insects11030182

**Published:** 2020-03-13

**Authors:** Richard W. Mankin, Rikin Patel, Mason Grugnale, Ethan Jetter

**Affiliations:** 1 Center for Medical, Agricultural, and Veterinary Entomology, US Department of Agriculture, Gainesville, FL 32608, USA; mgrugnale2113@gmail.com; 2 Department of Biology, University of Virginia, Charlottesville, VA 22904, USA; rap5hf@virginia.edu; 3 Department of Nutritional Sciences, University of Florida, Gainesville, FL 32611, USA; ejetter19@gmail.com

**Keywords:** mating behavior, citrus greening disease, aggregation

## Abstract

Adult *Diaphorina citri* (ACP) use visual and chemical cues to locate young citrus flush shoots on which they forage and oviposit, and they use vibrational communication duetting calls as cues to help locate mates. For individual pairs, calling and mating usually peaks between 10:00 and 15:00. To explore whether call rates (calls/h) are affected by interactions with nearby conspecifics, rates were compared in small citrus trees on which either 5 or 25 ACP female and male pairs had been released at 17:00 for later recording from sunrise (06:00) to 22:00. Final ACP locations were noted 40 h after release. Call rates were similar in both treatments during normal mating hours. However, rates were significantly higher for low- than high-density treatments between 06:00 and 10:00, which suggests calling during this period may be affected by conspecific density. Both sexes aggregated on flush at both densities. We discuss the potential that ACP producing calls near sunrise, outside of normal mating hours, might benefit from gains in reproductive fitness in low-density contexts if they call not only to locate mates but also to locate preferred flush—in which case, co-opting of vibrations to disrupt both mating and foraging may be feasible.

## 1. Introduction

Methods to co-opt forage- and mate-locating cues for management of the Asian citrus psyllid, *Diaphorina citri* Kuwayama (Hemiptera: Liviidae), are drawing increased focus to reduce its populations and its spread of the economically devastating huanglongbing disease in citrus groves [1,2]. Adults of both sexes locate host citrus trees through visual and olfactory cues [3] and have similar propensities to disperse onto new host trees when conspecific populations build up to levels that reduce reproductive fitness [4]. Once on the hosts, females seek young leaf-flush shoots [5] on which to feed and lay eggs, as nymphs are obligate flush feeders [6]. Adults and nymphs readily acquire the disease-causing bacteria from infected trees during feeding activity, and adults then transmit the bacteria to uninfected trees as they move about the grove [7].

The mechanisms by which *D. citri* locate flush shoots are not fully understood, but their positive phototropic behavior [8,9,10] assists in locating shoots on the outer branches, and the preference of females for young flush is known to be guided by assessments of tissue hardness [11,12] and chemical cues that vary with leaf age [13,14]. In addition, females are attracted to odors from feeding damage by conspecifics but, once on the tree, show preference for uninfested young flush in the presence of odors from feeding damage [15].

For mating, *D. citri* employ substrate-borne vibrational communication duets—during which a male first calls and then moves towards a replying female [16,17,18]. There is evidence that males are attracted to one or more cuticular hydrocarbons produced by females [19] and they may be stimulated to produce vibrational signals in the presence of female cuticular hydrocarbons [20]. Because males search for females with which to mate and also search for flush on which to feed, they often are found co-aggregated with females on young flush shoots [5,21].

Females have been observed to call in the absence of males, but the role of calls occurring outside of vibrational communication duets is not well understood [16,22]. Vibrational communication has also been employed by different hemipterans to locate food resources or protective aggregations of conspecifics [23,24], and it sometimes plays a role in male–male rivalry [25,26] or female–female rivalry [27]. Rivalry or aggressive behavior has not yet been observed in psyllids [28].

The effects of multiple nearby conspecifics on *D. citri* vibrational communication remain largely unexplored as, until now, interactions between duetting pairs of males and females alone on a tree have been a primary focus of study [16,17,18]. In single-pair studies, mating occurs primarily between the hours of 10:00 and 15:00 under natural light conditions but has been reported to occur at low rates from near sunrise until shortly after sunset [16,17,18,29]. A few recordings of multiple *D. citri* pairs have been collected from infested trees in field environments [30], but no experiments continuously recording calls over the full range of daylight hours have been reported. To investigate whether vibrational call activity [17] is differentially affected by increased numbers of nearby conspecifics, a study was conducted to compare the rates of calls/psyllid/h from 5 *D. citri* of each sex with the rates from 25 of each sex beginning at dawn after their release the previous afternoon onto one of two small trees of similar size and flush characteristics.

## 2. Materials and Methods

### 2.1. Bioassay Arena

Tests were conducted in a 200 by 200 by 206 cm bioassay chamber shielded by carpeting and acoustic foam for protection against sound and vibration interference. Two 10 cm square (575 cm^3^) pots—each containing a small *Citrus macrophylla* Wester (Sapindales: Rutaceae) tree, of approximately 15 cm in length—were selected from a greenhouse at the Center for Medical, Agricultural, and Veterinary Entomology (CMAVE), Gainesville, FL 32608, USA. Each tree was pruned regularly to stimulate production of ten flush shoots. One day before testing, each potted tree was placed on a metal stand on the floor of a 34 by 34 by 61 cm nylon-mesh bug dorm (Model 1466BV, BioQuip, Rancho Dominguez, CA, USA) sitting on a rack in the bioassay chamber. Light was provided by two 42 W LED panels (Model MXL-301, Utilitech, West Lawn, PA, USA), approximately 38 cm above the tops of the dorms, operating on a 14:10 light:dark cycle that began at 06:00.

### 2.2. D. citri Handling Procedures: Treatments at P_5_ and P_25_ Densities

On each of the 17 different test days, eighty mixed-age adult *D. citri* were aspirated from a colony in the CMAVE greenhouse [31], initiated before the ‘*Candidatus* Liberibacter Asiaticus’ bacteria causing huanglongbing was introduced into Florida and tested periodically for its continued absence [29]. They were chilled for 1 min in a freezer and sexed under a dissecting microscope. Five sexed adults each were placed in 12 petri dishes of the same sex. At 17:00, one dish with 5 males and one with 5 females were placed on the floor of the dorm designated as the *P*_5_ treatment and the remaining dishes with a total of 25 males and 25 females were placed on the floor of the dorm designated as the *P*_25_ treatment. Vibrational and video recording began at 06:00 the next morning. The surviving *D. citri* were removed and discarded on the day after recording after their locations and aggregational distributions were noted. The trees were rinsed with water, air-dried in the greenhouse, and pruned as needed before subsequent tests. The bug dorms were frozen and wiped down to eliminate insects, debris, and surface contamination before reuse. Treatments were rotated between trees after each test and the tree positions were rotated after each pair of tests to mitigate potential effects of tree and position within the chamber on *D. citri* behavior.

### 2.3. Recording Procedures: Treatments at P_5_ and P_25_ Densities

To record *D. citri* vibrational calls and incidental movements, an accelerometer (Model 352A24 PCB Piezotronics, PCB Technologies Ltd., Depew, NY, USA) was attached with beeswax to the lower stem of each tree. The accelerometer cable was extended through the zipper opening of the bug dorm to an amplifier (Model 480EO9, PCB Technologies Ltd.) with 20 dB gain. The amplifier signal was fed to an audio recorder (Model PMD661, Marantz, Cumberland, RI, USA) which stored the signals on a 32-GB SD memory card for subsequent signal analyses. The digitization rate was 44.1 kHz in 16-bit, mono format. Continuous recordings were collected from each tree between times of 06:00 and 22:00. The recordings were subdivided into 10 min segments to accommodate memory limitations of the desktop computer that processed the vibrational signals afterwards using Raven 1.5 (Cornell Laboratory of Ornithology, Ithaca, NY USA) [32]. Live visual observations were enabled by use of separate video camera recorders (model HDR-SR1, Sony Corp, New York, NY, USA) focused on each tree. The vibrational and video signals were fed to a monitor outside the bioassay chamber for observation without interference from incidental background noise.

**Figure 1:**
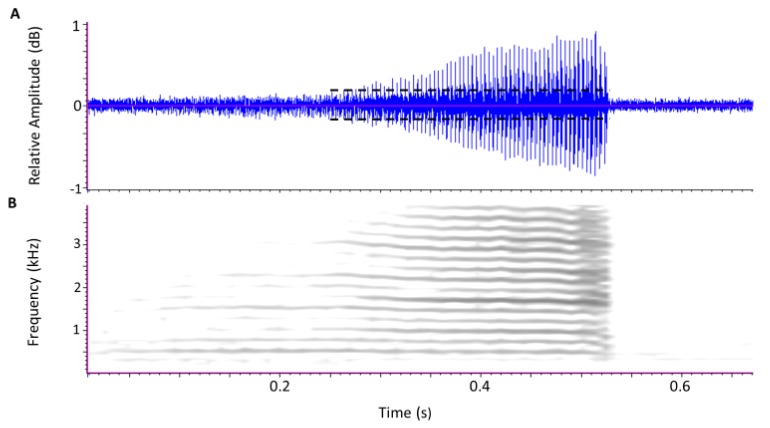
Oscillogram (**A)** and spectrogram (**B**) of a 0.28 s, *D. citri* vibrational call. Dashed lines indicate the threshold amplitude for background noise, above which the signal analyzer program checks for the presence of wingbeat pulses. Darker shades in the spectrogram (1024 point spectrum width) indicate greater energy at the specified time and frequency. The ca. 250 Hz wingbeats appear as closely spaced signal pulses in the oscillogram and display multiple harmonics of 250 Hz in the spectrogram (1024 point spectrum width).

The first stage of signal analysis involved visual and aural screening of each 10 min segment with Raven to identify potential intervals of calling as well as periods of movement or other background noise, including vibrations produced by the tree itself [33]. When listeners detected such intervals, the 10 min segment was further analyzed using an automated signal analysis program, DAVIS, described in (Mankin [34,35]), which searched consecutive digital samples of the recorded segment for signal pulses with magnitudes that rose above a specified background threshold, e.g., a wingbeat pulse (Figure 1). When the threshold was exceeded, DAVIS then entered a subroutine which examined a series of consecutive 5 ms sections, calculating a 512 sample spectrum centering on the peak amplitude of the section. The series continued until the peak magnitude failed to exceed the threshold for 200 ms. The complete set of signal pulses between the first and last signal magnitude above threshold was labeled as a pulse train.

**Figure 2:**
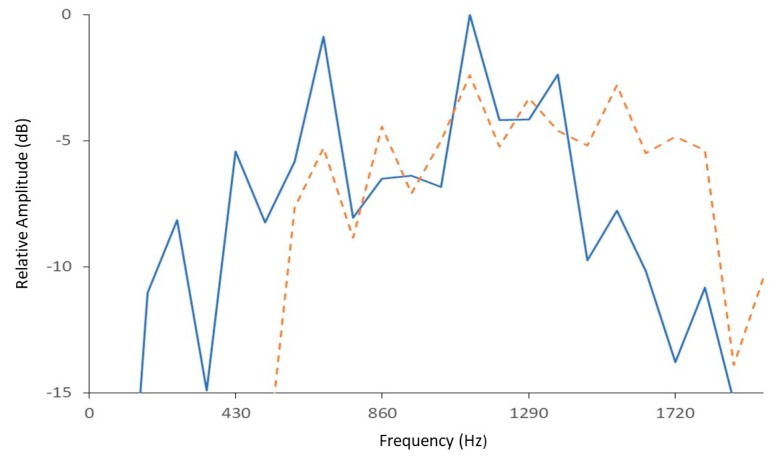
Profiles (mean spectra) of two representative vibrational calls (designated by solid and dashed lines) produced by *D. citri* on small citrus trees, recorded by methods described in Wenninger et al. [16]. The profiles were used to automate discrimination and counting of calls produced by *D. citri* in this study.

Each spectrum in the resultant pulse train was least-squares matched with two spectral profiles [34] of known *D. citri* calls described previously in [16] (Figure 2). Spectra that matched well with either or both profiles were counted as potential sections of a call, and poor matches were classified as noise. DAVIS then passed through the recording again, classifying pulse trains as *D. citri* calls if they contained seven or more sections and at least 50% of the spectra in the train matched a *D. citri* profile. The spectra of the wingbeat pulses in Figure 1, for example, matched well with both spectral profiles in Figure 2, as can be seen in the close matchups between the frequencies of harmonic peaks in the spectrogram of Figure 1 and the frequencies of spectral peaks of the two profiles of Figure 2.

**Figure 3:**
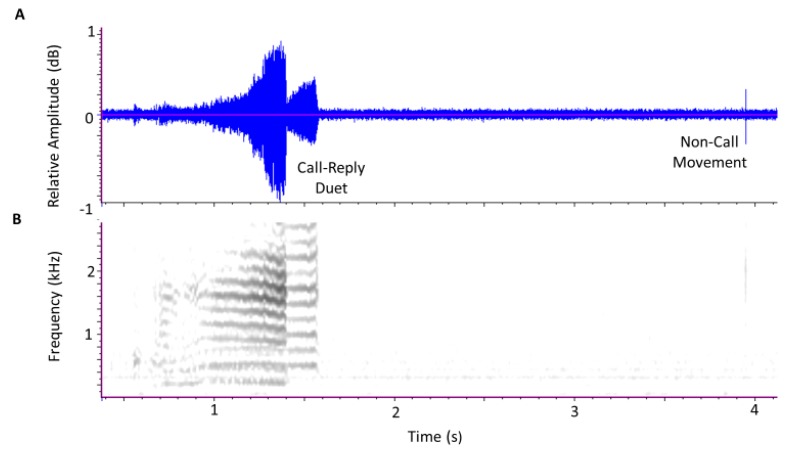
Oscillogram (**A**) and spectrogram (**B**) of a 4 s interval containing a *Diaphorina citri* call–reply duet and a non-call movement vibration. The spectra in the call–reply pulse train matched the *D. citri* profiles in Figure 2 but the spectra in the non-call pulse train failed to match either profile, and the pulse train was classified as a non-call movement.

Other pulse trains that included insect movements or background noise, as in the example of Figure 3, were classified as non-calls. In general, as in Figure 3, the non-call pulse trains were much shorter in duration than *D. citri* calls. The beginning and ending times of both calls and non-calls were saved in a spreadsheet. Non-calls were further distinguished by listener assessment as either insect movement or other background noise. The counts of calls obtained from the 10 min segments were then summed over 1 h periods to obtain the numbers of calls per hour. The recording period was subdivided into four different calling period intervals based on differences in *D. citri* behavior at different times of day after sunrise (06:00). The intervals were designated as (1) sunrise: 06:00–10:00, (2) mating hours: 10:00–16:00, (3) evening: 16:00–20:00, and (4) darkness: 20:00–22:00.

### 2.4. D. citri Handling Procedures: Flush Shoot Aggregation Sex Ratio Tests

**Figure 4:**
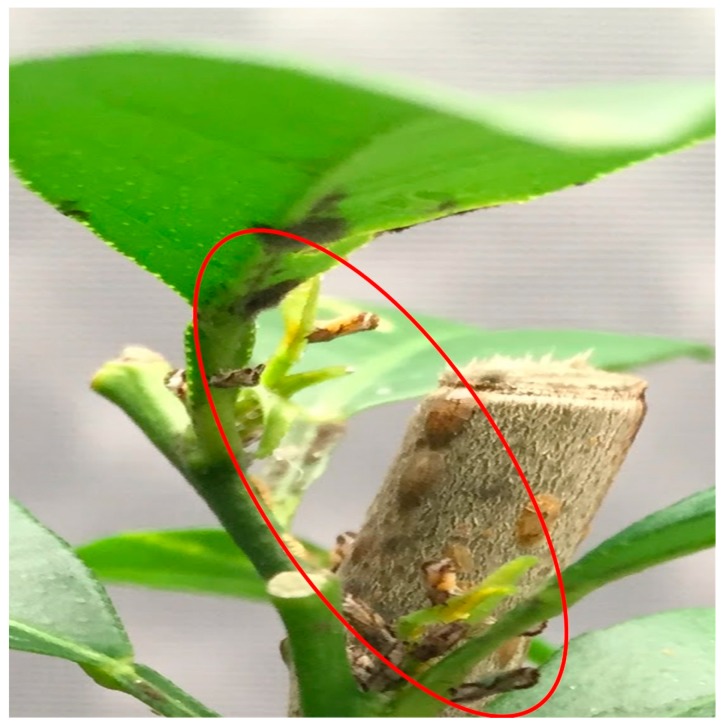
Example of multiple psyllids (circled) aggregating on young flush shoots at the end of a density treatment trial.

During the density treatments study, it was noted that psyllids were frequently aggregated in groups of four or more on flush shoots when they were removed from the trees at the end of each trial, approximately 40 h after the ACP were released onto the tree (Figure 4). The aggregations occurred more frequently on young than mature flush. This led to questions about potential interactions of males and females together on the same flush shoots that might lead to reductions in calling rates during mating hours. Additional studies were then conducted to consider the distributions of females and males in aggregations on young and mature flush.

Nine tests were conducted by releasing 25 psyllids of each sex into a bug dorm containing a small tree in the bioassay arena. Each test used the tree pruning procedure and the same release times and light cycle as in Section 2.1. On the morning of the second day, approximately 40 h after the insects were released, a flush shoot containing an aggregation of at least 4 psyllids was transferred into a vial, chilled for 1 min in a freezer, and sexed under a dissecting microscope. The counts for each aggregation were tabulated.

### 2.5. D. citri Handling Procedures: Flush Age Tests

Ten tests were conducted similarly to those in Section 2.4 except that, at the end of each test, all flush shoots containing aggregations of four or more psyllids were placed into vials. The psyllids were chilled for 1 min in a freezer and sexed under a dissecting microscope, and the dimensions of the flush materials were measured on a millimeter-scaled grid to estimate the surface area of the plant matter on which the aggregation occurred. The flush then was divided into two groups based on size: young flush with leaf surface area <2.5 cm^2^ and mature flush with area >2.5 cm^2^. The category of young flush corresponded approximately to categories SI and SII in [12], and categories V1–V3 in [36]. The areal density of females and males per flush shoot was calculated as the number of females and males in the aggregation divided by the estimated surface area in cm^2^.

### 2.6. Statistical Analyses

Shapiro–Wilk tests (Univariate Procedure, SAS Version 9.4 [37]) were performed to determine the normality of the distributions of call rates (calls/psyllid/h) used for analyses of the *P*_5_ and *P*_25_ psyllid density treatments over time. Because the distributions were found not to be normal (N = 68, W= 0.646, *p* < 0.001 for the *P*_5_ tests, and N = 68, W = 0.379, *p* < 0.001 for the P_25_ tests), the rates were transformed as log10 (rate+1). A sphericity test was conducted using Proc GLM [37] (df = 5, Mauchly’s Criterion = 0.887, χ^2^ =3.70, *p* = 0.594), which indicated the variances of the differences between transformed call rates for all possible pairs of days were not significantly different, enabling repeated measures analysis of variance of call rates to be conducted (Table 1). A Tukey Honest Significant Difference (HSD) test [37] was conducted for comparisons between *P*_5_ and *P*_25_ calling rates during different calling period intervals.

Differences in the occurrence of calls during different calling period intervals in the *P*_5_ and *P*_25_ density treatments were compared by enumerating the numbers of test days in which calls were detected during a specified calling period interval. Counts of the numbers of days in which calls were detected in the sunrise, mating period, evening, and darkness intervals during the 17 days of testing were compared between treatments by χ^2^ tests.

Shapiro–Wilk tests were conducted to determine the normality of the distributions of females and males counted on flush samples and the normality of the distributions of female and male densities on young and mature flush. Based on the results, a Student’s t test was conducted to compare the distributions of female and male counts, and a non-parametric Mann–Whitney U test was used to compare the mean densities of females and males on young and mature flush.

## 3. Results

### 3.1. Calls and Movement Vibrations in Treatments at P_5_ and P_25_ Densities

Vibrational calls occurred in all 17 days of testing, in addition to vibrations with spectral and temporal features like those documented as crawling or scraping in previous studies [38]. Loud buzzing and jumps from the tree occurred in several tests. The frequent occurrence of crawling and scraping movements was confirmed by inspection of the video recordings, which showed additional movements that were too weak to be detected as vibrations by the accelerometer. For this reason, we did not quantify the numbers of movement vibrations, but considered them an indication that the psyllids frequently walked along the branches during both lights-on and light-off cycles.

### 3.2. Calling Rates in Treatments at P_5_ and P_25_ Densities

**Figure 5:**
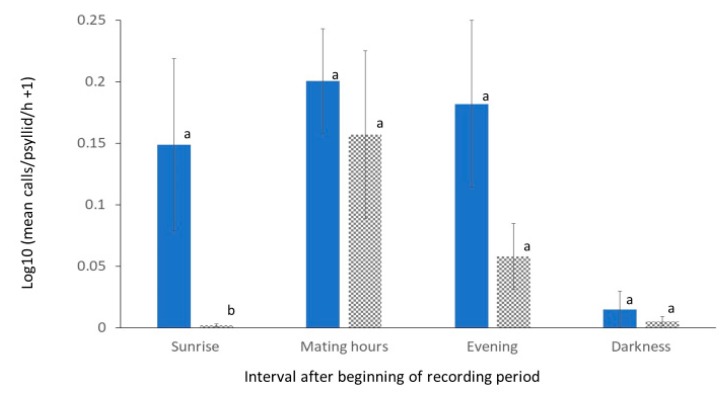
Log10-transformed means and standard errors (SEs) of the number of vibrational calls/psyllid/h detected over time after sunrise in tests with 5 (solid bar) or 25 (checkered bar) *D. citri* female and males released onto small citrus trees. Interval time periods were sunrise: 06–10:00, mating hours: 10–16:00, evening: 16–20:00, and darkness: 20–22:00. The SE are marked by capped-lines. Call rates in each recording period interval marked with the same letter are not significantly different (*p* > 0.05 in a Tukey HSD test).

The rates of vibrational calls varied considerably over time in both treatments, with the highest rates occurring during sunrise, mating hours, and evening intervals but few during darkness (Figure 5).

Analysis of variance of calling rates indicated statistically significant effects of psyllid density treatment and calling period intervals, but not the interaction between density and calling period interval (Table 1). The differences were not statistically significant except during the sunrise calling interval—during which, the calling rate was significantly greater for the *P*_5_ than the *P*_25_ treatment. There was no significant effect of density on the rates of calling during mating hours at the tested levels of population density.

Overall, 58 of the 1428 10 min periods in the experiment contained calls in the *P*_5_ treatments, and 26 contained calls in the *P*_25_ treatments. The numbers of test days with calls were not significantly different between treatments except in the sunrise calling period interval, where 8 days contained calls at in the *P*_5_ treatment and 2 in the *P*_25_ treatment (df = 1, *χ*^2^ = 5.10, *p* = 0.029). A mean of 8 psyllids were retrieved from the trees at the end of each test in the *P*_5_ treatments and 34 in the *P*_25_ treatments.

### 3.3. Distributions of Females and Males in Flush Aggregations

A mean of 4.8 ± 2.49 standard error (SE) females per aggregation sample and 2.6 ± 1.33 males were obtained in tests to consider the sex ratio in psyllid aggregations, a female to male ratio of 1.8. Because a Shapiro–Wilk test had indicated that the distributions of counts were not significantly different from normal (female W = 0.90, n = 9, *p* = 0.24; male W = 0.88, n = 9, *p* = 0.15), a Student’s t test was conducted, which found that the mean number of females per aggregation was significantly different from the mean for males (*p* = 0.019).

In tests comparing the numbers of psyllids per unit surface area (areal density) on young and mature flush, a Shapiro–Wilk test indicated that the counts were not distributed normally for either females (W = 0.925, n = 39, *p* = 0.013) or males (W = 0.92, n = 39, *p* = 0.009). Likewise, the Shapiro–Wilk test indicated that the areal densities were not distributed normally for either females (W = 0.777, n = 33, *p* < 0.001) or males (W = 0.841, N = 33, *p* < 0.001). The mean density for both females and males on young flush was significantly greater than on mature flush (Table 2). However, although the density of females was higher than that of the males on young flush (Score = 80.5, U = −1.81, *p* = 0.07) and mature flush (Score = 568.5, U = 0.60, *p* = 0.55), the differences between densities of females and males on each flush type were not statistically significant. It should also be noted that, overall, 56% of psyllids released in the flush age treatments were retrieved in aggregations containing four or more psyllids.

## 4. Discussion

The results indicate that the density of *D. citri* populations on host citrus trees affects the timing and rates of their duetting call behaviors. The distribution of call rates in Figure 5 is consistent with previous reports [22,29] that calling frequently occurs among single pairs between 10:00 and 15:00, but this study documents additionally that calling can occur between sunrise (06:00) and 10:00, particularly at the lower, *P*_5_ density. A rationale for such a change in the pattern of calling in this context is suggested by considering that successful foraging on young flush, an ephemeral resource (with only approximately 18 d from bud swelling to maturity [36]), is a strong contributor to both female and male reproductive fitness [12]. Mating behavior [29] and foraging [4] both often occur on flush, and young flush is preferred by both sexes for foraging. Consequently, there is potential that calling behavior occurring outside of the peak mating period on trees with low-density *D. citri* populations can play a beneficial role not only to help locate mates but also to help both sexes locate preferred forage.

*Diaphorina citri* is known to respond strongly to visual cues when dispersing, and dispersal flights in field environments usually begin at approximately 10:00 [39]. While on a citrus host, however, if either sex is seeking to disperse to young flush because they have not detected it through visual or olfactory cues, calling by either a male or a female might elicit a reply from a female already present on young flush, which would assist the caller in locating a site of preference. Such behavior might explain the role of occasional calls by individual *D. citri* females in the absence of a duetting male [16]. The female may be calling primarily to obtain information about the location of nearby young flush. Although females usually do not move when they respond to males during duetting behavior, they have been observed to move in the context of foraging [17,18]. The finding that 56% of the psyllids released in the flush aggregation tests (Section 3.3) were found in aggregations on flush on the day after their release supports the possibility that calling by either female or male *D. citri* followed by movement towards the source of a reply might lead the caller to flush. Similar behaviors have been reported in other hemiptera [23,24]. Flush-seeking behaviors in *D. citri* are known to be density dependent, however. *Diaphorina citri* females show less preference to flush associated with odor from 10 or more females [15]. Density dependent effects might lead to reduced levels of calling to locate new foraging sites when females are present on a tree at high density. At low density, however, it could be beneficial to begin searching for flush on the host tree as soon as dawn, when it becomes possible to see a conspecific and the quality of the flush from which the conspecific has replied after a call.

Other instances have been observed where male insects that communicate through vibrational calls modify the spectrotemporal patterns of their calls in the physical presence of other males [40]. Thus, it is not completely unprecedented that density dependent effects might occur in different vibrational communication contexts and could contribute to the differences observed in this study between the *D. citri* calling rates at sunrise in the *P*_5_ and *P*_25_ density treatments. It remains to be determined whether calling outside of the mating hours interval is primarily by one sex and whether hunger or other physiological states also affect the likelihood that a psyllid will call.

Like the calling behaviors noted above that may be beneficial for both foraging and mating, call-fly behaviors previously identified by Hunt and Nault [41] also might lead male *D. citri* fortuitously to both a potential foraging site and a mating site after they have just migrated to a new tree. In previous reports where females and males at sampling sites have been counted, a slight preponderance of females has been found [42,43,44], which suggests that males would benefit by using calling behavior for either mate seeking or foraging. It is thus not surprising that mate-seeking calling behavior might evolve new roles or that individual *D. citri* in the presence of small numbers of conspecifics might behaviorally adapt through associative learning to call for help in locating preferred foraging sites [45,46,47,48]. Alternatively, a conspecific that would benefit from the presence of other conspecifics might call to recruit conspecifics [49].

Playback of hemipteran vibrational communication signals has been shown to trap males or have disruptive effects on mating success in multiple lab and field studies [19,27,30,50,51,52,53,54,55,56,57]. Thus, it may be worthwhile to consider the potential of vibrational signals that could disrupt foraging as well as mating in *D. citri*.

Finally, it has been suggested that reproductive fitness of *D. citri* is also affected by the presence of the *Candidatus* Liberibacter asiaticus bacterial agent that causes huanglongbing [58]; consequently, in field environments where the disease vector is present, the foraging and mating behavior might be influenced by the fraction of infected psyllids. Further studies are needed to resolve these and other unknowns about the mating and foraging interactions among female and male *D. citri* at different population densities.

## 5. Conclusions

This investigation about the effects of *D. citri* population density on vibrational communication signals and the formation of aggregations on citrus flush contributes to understanding *D. citri* female and male mating and foraging behaviors in different contexts. It also raises questions that suggest the need for additional study. As expected, calling occurred at the highest rates during the normal mating hours, independent of population density. However, calling occurred at significantly higher rates during the sunrise calling period at the *P*_5_ density compared to the *P*_25_ density. The known tendency of *D. citri* to aggregate on citrus flush [5,21] was observed at both densities, suggesting that neither of the tested densities was sufficiently high to reduce the beneficial effects that aggregation on citrus flush has on reproductive fitness. In studies to consider aggregations on different kinds of flush, females were found to aggregate most densely on the youngest flush. Females were present at greater densities than males on young and mature flush, but the differences were not statistically significant. Efforts to co-opt forage- and mate-locating vibrational cues for *D. citri* management would likely benefit from further study of the density dependent behaviors observed in this exploratory report.

## Figures and Tables

**Table 1:** Repeated measures analysis of variance for psyllid density, calling period intervals, and interaction between calling period interval and density in *P*_5_ and *P*_25_ psyllid density treatments.

Source	df	Mean Square	*F*	*p*
Psyllid density	1	0.224	5.06	0.031 *
Calling period interval	3	0.172	4.76	0.039 *
Interval–density interaction	3	0.036	1.00	0.390

*F* value is significant at the * *p* < 0.05 level.

**Table 2:** Mean ± SE of female and male *D. citri* areal densities on young and mature flush.

Sex	Areal Density (No. psyllids/cm^2^) On Flush Type		Score	U	*p*
	Young (n = 12)	Mature (n = 23)			
Female	2.72 ± 0.42	0.41 ± 0.07	280	4.29	<0.0001
Male	1.60 ± 0.29	0.47 ± 0.08	252	3.19	0.0013
